# Identification and Functional Analysis of microRNAs Involved in the Anther Development in Cotton Genic Male Sterile Line Yu98-8A

**DOI:** 10.3390/ijms17101677

**Published:** 2016-10-07

**Authors:** Xiaojie Yang, Yuanming Zhao, Deyi Xie, Yao Sun, Xunlu Zhu, Nardana Esmaeili, Zuoren Yang, Ye Wang, Guo Yin, Shuping Lv, Lihong Nie, Zhongjie Tang, Fu’an Zhao, Wu Li, Neelam Mishra, Li Sun, Wei Zhu, Weiping Fang

**Affiliations:** 1 Economic Crop Research Institute, Henan Academy of Agricultural Sciences, Zhengzhou 450002, China; yyxxjj7910@sina.com (X.Y.); zzzym5@sina.com (Y.Z.); xiedeyi101@sina.com (D.X.); yangming1697@sina.com (Y.S.); lvshuping316@sina.com (S.L.); nlh200999@sina.com (L.N.); tangzhongjie2001@sina.com (Z.T.); fazcotton@sina.com (F.Z.); cotton169@sina.com (W.L.); 2 Department of Biological Sciences, Texas Tech University, Lubbock, TX 79409, USA; xunlu.zhu@ttu.edu (X.Z.); nardana.esmaeili@ttu.edu (N.E.); neelam.mishra@ttu.edu (N.M.); li.sun@ttu.edu (L.S.); 3 State Key Laboratory of Cotton Biology, Cotton Research Institute, CAAS, Anyang 455000, China; yangzuoren4012@163.com (Z.Y.); wangye_916@163.com (Y.W.); 4 Handan Agronomy of Agricultural Sciences, Handan 056006, China; yinwenjie7514053@sina.com; 5 Agronomy College, Henan Agricultural University, Zhengzhou 450002, China; zhuwei_2006z@sina.com

**Keywords:** cotton, male abortion, miRNAs, targets, molecular regulation, phytohormone

## Abstract

Hybrid vigor contributes in a large way to the yield and quality of cotton (*Gossypium hirsutum*) fiber. Although microRNAs play essential regulatory roles in flower induction and development, it is still unclear if microRNAs are involved in male sterility, as the regulatory molecular mechanisms of male sterility in cotton need to be better defined. In this study, two independent small RNA libraries were constructed and sequenced from the young buds collected from the sporogenous cell formation to the meiosis stage of the male sterile line Yu98-8A and the near-isogenic line. Sequencing revealed 1588 and 1536 known microRNAs and 347 and 351 novel miRNAs from male sterile and male fertile libraries, respectively. MicroRNA expression profiles revealed that 49 conserved and 51 novel miRNAs were differentially expressed. Bioinformatic and degradome analysis indicated the regulatory complexity of microRNAs during flower induction and development. Further RT-qPCR and physiological analysis indicated that, among the different Kyoto Encyclopedia Gene and Genomes pathways, indole-3-acetic acid and gibberellic acid signaling transduction pathways may play pivotal regulatory functions in male sterility.

## 1. Introduction

Cotton is an important economic crop for its natural source of fiber, as well as high-quality protein and oil [1]. Cotton heterosis has the potential of increasing yield from 10%–20% and could therefore significantly impact breeding programs, if it could be appropriately exploited. Although genic male sterility (GMS) has been widely used in the utilization of hybrid vigor based on its unique advantages, the complex regulatory network of GMS occurrence has yet to be extensively investigated.

MicroRNAs (miRNAs) are a class of endogenous small regulatory non-coding RNAs, which negatively regulate the expression of target genes at the post-transcriptional level through degrading target mRNAs or repressing gene translation [2]. miRNAs may also regulate the expression of target genes through DNA methylation [3] and function to regulate multiple developmental processes, including seed germination [4], root development [5], leaf development and polarity [6], and floral organ identity [7,8]. In addition, miRNAs are also involved in plants’ responses to diverse environmental biotic and abiotic stresses, such as drought [9], salinity [10] and pathogen invasion [11].

Different miRNAs serve regulatory functions in floral development in various plants. In *Arabidopsis*, miR172 causes early flowering and disrupts floral organ identity through regulation of the *AP2* gene via a translational mechanism [7,12]. The SBP-box gene *SPL8*, which is essential for proper development of sporogenic tissues by regulating genes mediating cell division, differentiation and specification in early anther development in *Arabidopsis*, is regulated by miRNA 156/7 [13]. In rice, OsmiR396d controls spikelet development by regulating the expression of the *OsGRF* gene, which functions with OsGIF1 in floret development through targeting of *JMJ706* and *OsCR4* genes [14]. miR159 indirectly affects the development of aleurone cells and flowers via regulation of a *GAMYB* gene, which plays an essential role in gibberellin signaling, in rice [15]. In addition, miR159 is also involved in the expression of the *DUO1* gene, which is required for cell division in male gametophytes [16]. During auxin signaling, miR160 directs the post-transcription regulation of the auxin response transcription factor *ARF17*. Changes in the expression of *ARF17* lead to many developmental defects, including abnormal stamens, sterility and premature inflorescence development [17]. Similarly, two other auxin response transcription factors *ARF6* and *ARF8*, which regulate both stamen and gynoecium maturation, are regulated by miRNA167 in *Arabidopsis* [18,19].

The expression pattern of miRNA in several important male sterile crops, using high-throughput sequencing or microRNA array technologies combined with quantitative real-time PCR, suggests a complex multiple regulatory network of miRNAs. Upregulation of some miRNAs was essential for pollen abortion in a maize cytoplasmic male sterile line [20]. During the rice anther development process, the differential expression patterns of a total of 24 miRNAs were also identified in the cytoplasmic male sterile line MeixiangA [21]. In addition, similar results were also reported in *Brassica juncea* [22]. Comparative expression profiling of miRNA during anther development of cotton revealed that the target genes of miRNAs were expressed differentially between a GMS mutant line and the wild-type lines and were specifically involved in anther development [23]. Recently, several similar results have been reported in *Brassica campestris* ssp. *Chinensis* [24,25], radish [26] and soybean [27].

In the present study, high-throughput sequencing, combined with degradome sequencing, RT-qPCR and histological and biochemical analyses, was applied to identify and characterize the key miRNAs involved in the network underlying the abortion process of a novel male sterile mutant line Yu98-8A, in which the sterility is controlled by a single recessive gene (named *yms1*) and also displays a strong heterosis in various combinations. In this study, two independent small RNA libraries of the young buds collected from the pollen mother cell formation to meiosis stages were constructed based on the microscopic observation data. One thousand five hundred eighty eight and 1536 known miRNAs and 347 and 351 novel miRNAs were identified and predicted, and among them, 49 known and 51 novel miRNAs were differentially expressed. Targets analysis of differentially-expressed miRNAs indicated the regulatory complexity of miRNAs during flower induction and development. Further, RT-qPCR and physiological analysis indicated that, among the different KEGG pathways, indole-3-acetic acid (IAA) and gibberellic acetic acid (GA) signaling transduction pathways may play pivotal regulatory functions in male abortion.

## 2. Results

### 2.1. Morphological and Histological Identification of Male Genic Sterile Buds

**Figure 1:**
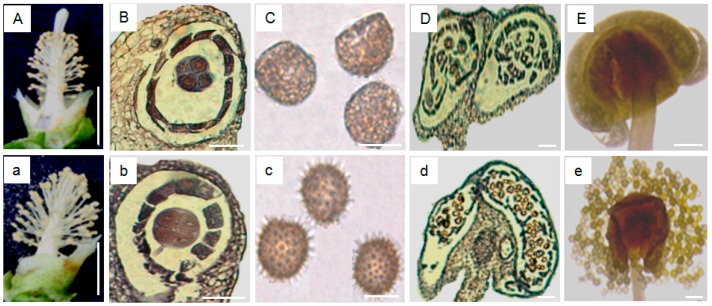
Microscopic analysis of flower development in genic male sterile mutant (MS, upper) and normal wild-type (male fertile (MF), lower) flowers. (**A**,**a**) The longer exposed stigma and normal phenotype of MS and MF flowers, respectively. (**B**–**E**) The tetrad of microspore of meiosis, pollen grains, developing anther and the final empty anther of MS buds, respectively. (**b**–**e**) correspondingly represent the different stages of MF buds, respectively. Bars = 1 cm in (**A**,**a**), 50 μm in (**B**,**b**,**C**,**c**), 200 μm in (**D**,**d**) and 500 μm in (**E**,**e**), respectively.

Wild male fertile (MF) flowers displayed a normal floral phenotype in the days post-anthesis (DPA) (Figure 1a), while the genic male sterile mutant (GMS) showed an abnormal longer exposed stigma (Figure 1A). No significant phenotypic differences between MS and MF buds were observed in the sporogenous cell formation (GMS: ≤1.8 mm; MF: ≤2.0 mm) or pollen mother cell formation (GMS: 1.8–2.5 mm; MF: 2.0–2.8 mm) stages. However, during meiosis, especially in the tetrad formation stage (MS: 2.5–3.0 mm; MF: 2.8–3.3 mm), the GMS line Yu98-8A showed a shriveled tetrad, and no spinescent protuberances on the pollen wall were detected (Figure 1B,C), but MF flowers had normal tetrad and pollen morphologies (Figure 1b,c). Furthermore, abnormal and fragmented microspores were observed in Yu98-8A buds, apparently resulting in the formation of empty anther sacs due to eventual pollen degradation (Figure 1D,E). In contrast, wild-type anther sacs contained large numbers of pollen grains (Figure 1d,e). The shriveled tetrad appeared to be the first significant observable feature of the abnormal MS phenotype, indicating that the pollen mother cell formation phase and the following meiotic cycle were early indicators of microspore abortion in Yu98-8A.

### 2.2. Analysis of the Small RNA Library and Expression Profile of Small RNAs during Anther Development

**Figure 2:**
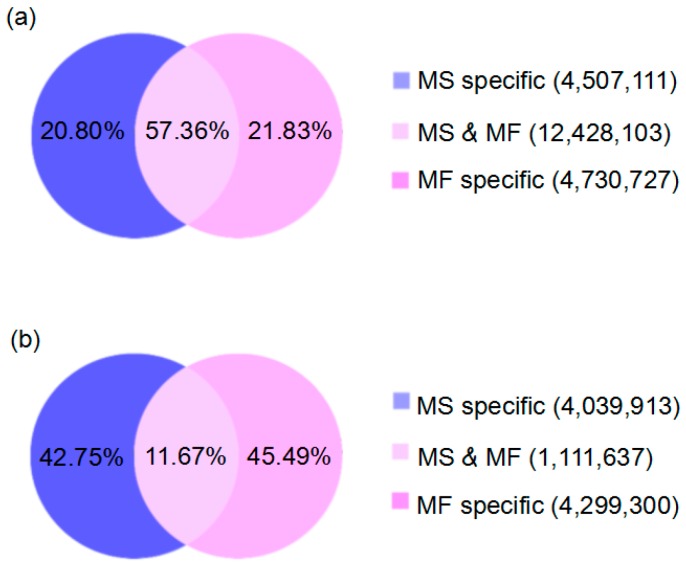
Summary of specific and common clean reads between two independent MS and MF libraries. Venn charts (**a**,**b**) show the distribution of the total small RNAs and the unique small RNAs between the MS and MF libraries, respectively.

To investigate the miRNAs involved in microspore abortion in cotton GMS mutant line Yu98-8A and the corresponding MF type, two independent small RNA libraries of the young buds collected from the pollen mother cell formation to meiosis stages were analyzed based on the microscopic observation data using Illumina Solexa high-throughput sequencing technology. The MS and MF libraries generated 11,095,604 and 10,741,527 raw reads, respectively. In general, the sequence length distributions of raw data were very similar between the two libraries. For example, the size of the majority of the small RNAs ranged from 21–24 nt in size in both MS and MF young buds, and small RNAs with 24 nt were the most abundant small RNAs in all sequence reads (Figure S1). After removing low quality reads including adapter and insert contaminants, RNAs shorter than 18 nucleotides and polyA, 11,002,282 and 10,663,659 clean reads were obtained, from the MS and MF libraries, respectively (Table S1). Further analysis of the variation of small RNA expression profiles in both MS and MF buds showed that, in addition to the common small RNAs accounting for 57.36% between the two libraries, the MS and MF libraries are comprised of 42.75% and 45.49% of the unique small RNAs, respectively (Figure 2b). The results indicated that up to 78.43% of the unique small RNAs in MS buds were expressed specifically in those buds.

To further annotate the small RNAs, all sequences corresponding to known non-coding RNAs, such as rRNAs, tRNAs, snRNAs and snoRNAs, possible degraded fragments of mRNA, repeat sequences and some non-annotated small RNAs were filtered. Finally, a total of 15,511 and 15,476 unique small RNAs were obtained from the MS and MF libraries, respectively, and these RNAs were used for the identification and prediction of conserved and novel miRNAs (Table 1).

### 2.3. Identification and Prediction of Known miRNAs and Novel miRNAs

To identify the miRNAs in young buds from the pollen mother cell formation phase and the following meiotic cycle, a total of 15,511 and 15,476 filtered unique small RNAs were identified by deep sequencing and compared with the currently-known mature plant miRNAs in miRBase. As a result, 1588 and 1536 known miRNAs, 206 and 205 miRNA-5ps, 165 and 167 miRNA-5ps and 1987 and 1842 hairpins belonging to 19 families were identified from the MS and MF libraries, respectively (Table S2).

**Figure 3:**
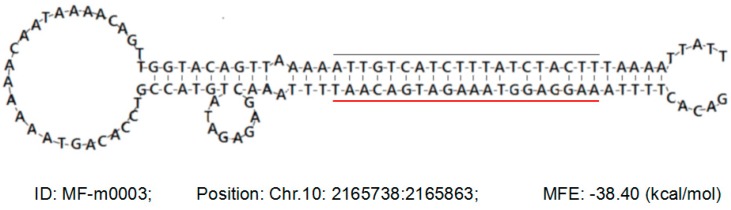
The predicted novel miRNA (MF-m0003) and the secondary hairpin structure of the corresponding precursor. The novel miRNA sequence (located on Chr.10: 2165738-2165863) (red underlined) and it’s complementary sequences (black line) in the hairpin structure, and the information including the position and minimum free energy (MFE) of the precursor are presented.

For further identifying potentially novel miRNAs, the cotton transcript assembly database (Available online: http://occams.dfci.harvard.edu/pub/bio/tgi/data/Gossypium) was chosen to map unique small RNA sequences, and the flanking genome sequences of unique small RNAs were used to predict the secondary hairpin structures and the minimum free energy by using MIREAP, a tool used to identify both known and novel microRNAs from small RNA libraries deeply sequenced by Solexa/454/Solid technology (Available online: http://sourceforge.net/projects/mireap/). Any small RNAs that met the certain criteria described in the Materials and Methods perfectly and exactly mapped to the genome sequence, but not the known plant miRNAs, were classified as novel miRNAs. As a result, a total of 347 and 351 novel miRNAs and their corresponding precursors were predicted from MS and MF young buds, respectively (Tables S3 and S4). As one example, one novel mature miRNA sequence miRNA and the secondary hairpin of the precursor are presented here (Figure 3).

### 2.4. Differentially-Expressed Analysis and Target Prediction of Known and Novel miRNAs

**Figure 4:**
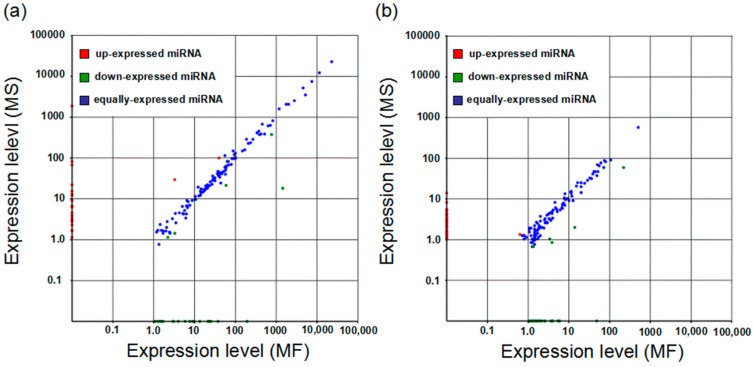
Scatter plot of differentially-expressed miRNAs between the two independent MS and MF libraries. (**a**,**b**) The conserved and predicted novel miRNAs, respectively. Red dots represent miRNAs with a fold change >2; blue and green dots represent miRNAs with 1.2 ≤ fold change <2 and with a fold change <1.2 (*p*-value < 0.05), respectively. The dots on the *X* and *Y* axes represent the specific expression of miRNAs in MF and MS young buds, respectively.

To identify the relative differential expression levels of conserved and novel miRNAs, the fold change and *p*-value were calculated to determine the significance of differential expression. The miRNAs with a fold change >2 (*p*-value < 0.05) and a fold change <1.2 (*p*-value < 0.05) were regarded as differentially-expressed miRNA. As a result, 49 known miRNAs, including 22 specific to MS, 20 specific to MF and 7 common miRNAs, were identified. For the novel miRNAs, 51, 39 and 4 novel miRNAs were expressed specifically in MS, MF and both samples, respectively (Figure 4 and Table S5).

In order to elucidate the functions of the differentially-expressed miRNAs identified in the young buds from the mother pollen formation to meiotic stages, target genes were predicted initially by bioinformatics. In addition, 100 and 281 different targets were predicted for 13 of 49 differentially-expressed known miRNAs and for 30 of 51 novel miRNAs. The prediction also showed that some miRNAs had multiple targets (e.g., miR160a targets genes Cotton_D_10032280/Cotton_D_10025306/Cotton_D_10019554/Cotton_D_10010678/Cotton_D_10008456/Cotton_D_10005922/Cotton_D_10006397/Cotton_D_10002224/). Furthermore, a single gene may potentially be targeted by several miRNAs (e.g., the gene Cotton_D_10036714 was targeted by novel_mir_137/23/270/323), which indicated the complexity of miRNA regulation (Table S6). Further KEGG analysis of all of the annotated target genes showed that these genes were mainly involved in ascorbate and aldarate metabolism, plant hormone signal transduction, metabolic pathways and starch and sucrose metabolism.

### 2.5. Experimental Identification of miRNA Target Genes by Degradome Analysis

**Figure 5:**
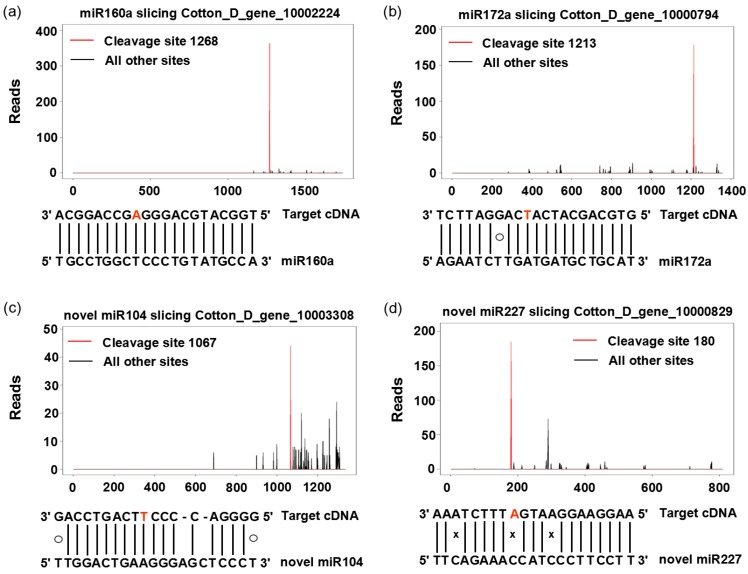
Target plots (t-plots) of miRNA targets confirmed by using degradome sequencing. Two degradome cDNA libraries were constructed from MS and MF young buds. t-plots (top) and the corresponding mRNA: miRNA alignment (bottom) is shown for each target transcript of two known (**a**,**b**); and two novel miRNAs (**c**,**d**). In each t-plot, the red line indicates the miRNA-directed cleavage of the target transcript. The solid line, the “O”, and “x” in miRNA:mRNA alignments indicate matched RNA base pairs, AC and GU mismatch, respectively. The red A and T represent the cleave sites of mRNA targets. The *X* axis indicates the nucleotide cleavage site, and the *Y* axis indicates the number of cleaved transcripts.

In order to provide further experimental evidence of the targets of differentially-expressed miRNAs and acquire more concrete targets, a degradome analysis was performed on the basis of bioinformatics prediction. A total of 222 annotated candidate target genes with 254 locations in the cotton genome of 70 known and novel miRNAs were detected, and most of these target genes were transcription factors, auxin response factors, TIR-NBS-LRR-like disease-resistant proteins, etc. (Table S7). As expected, the identified targets by degradome analysis were similar to those predicted by bioinformatics. For example, the identified targets of miR160a were the same as those by bioinformatics. However, some miRNAs had different targets from that predicted by bioinformatics (Table 2 and Table S7). The cleavage sites of the identified miRNA targets were presented in the form of target plots (t-plots), and in each of these t-plots, a clear peak for the absolute number of tags is found at the predicted cleavage site (Figure 5).

Further analysis of differentially-expressed miRNAs (Table S5) showed a total of 42 targets of five known miRNAs (17%, 5/30) and five targets of three novel miRNAs (23%, 3/13). Among the targets, auxin response factors (miR160a), DNA-directed RNA polymerases and NBS-LRR resistance protein-like proteins (miRNA2118a_3p), F-box family protein (miRNA394a), TCP4 (novel miRNA104) and chromatin licensing and DNA replication factor (novel miRNA20) were detected (Table 2).

### 2.6. Expression Pattern Analysis of miRNAs by RT-qPCR

**Figure 6:**
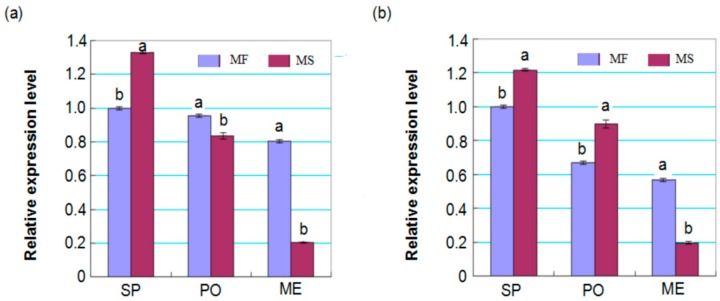
Expression pattern analyses of miRNAs of MS and MF young buds by RT-qPCR. (**a**,**b**) The expression profiles of miRNA159 and miRNA172, respectively. Relative expression was calculated by normalizing to the level of 5S rRNA in RT-qPCR. SP, PO and ME represent the sporogenous cell, pollen mother cell formation and meiotic stages, respectively. Bars represent means ± standard deviations, which were derived from three biological replicates (*n* = 3), and different letters above the bar indicate significant differences at *p* < 0.05.

Considering the fact that the small RNA libraries were constructed from a mix of different young buds, the differences of miRNAs’ expression between the MS and MF buds of certain developmental stages were likely eliminated. RT-qPCR analysis indicated that miRNA159 and miRNA172 displayed a low expression from the sporogenous cell formation to meiotic stage. Although the expression of both miRNAs in MS buds was far below that in MF buds, the expression of the two miRNAs in MS buds was 1.33- and 1.21-times higher than that in MF buds during the sporogenous cell formation stage (Figure 6), which indicated that the expression level of miRNA159 and miRNA172 plays important functions during the sporogenous cell formation stage in male sterile line Yu98-8A.

**Figure 7:**
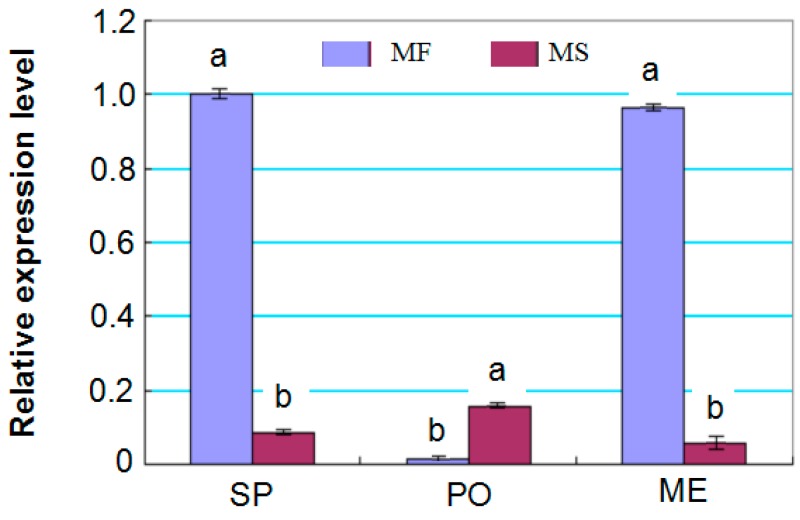
Expression pattern analysis of miRNA5658 of MS and MF young buds by RT-qPCR. Relative expression was calculated by normalizing to the level of the 5S rRNA coding gene in RT-qPCR. SP, PO and ME represent the sporogenous cell, pollen mother cell formation and meiosis stages, respectively. Bars represent means + standard deviations, which were derived from three biological replicates (*n* = 3), and different letters above the bar indicate a significant differences at *p* < 0.05.

In addition, although miRNA5658 has not been specifically identified (all *of* the *p*-values ≥ 0.05) by degradome analysis, it is noteworthy that it showed a very significantly higher expression in MF (Table S5). Furthermore, RT-qPCR analysis showed that during the sporogenous cell formation and meiosis stages, the expression of miRNA5658 in MF buds was about 11.7- and 16.2-fold higher than in MS buds, respectively (Figure 7). Combined with the target prediction by informatics (Table S6), it was supposed that miRNA5658 is involved in phytohormonal metabolism and signaling pathways during cotton flower development.

### 2.7. Dynamics of Indole-3-Acetic Acid Levels in Young Buds during Different Developmental Stages

**Figure 8:**
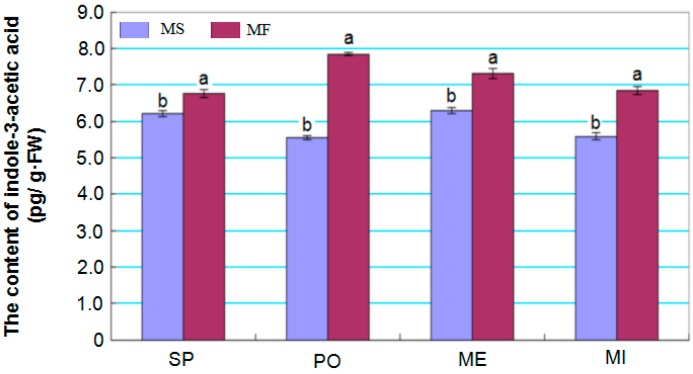
Indole-3-acetic acid (IAA) levels in young MF and MS buds. Data represent the mean values and standard errors from three replications during the sporogenous cell (SP), pollen mother cell formation (PO), meiosis (ME) and microspore development (MI) stages. Bars represent means ± standard deviations, which were derived from three biological replicates (*n* = 3), and different letters above the bar indicate a significant differences at *p* < 0.05.

Given that plant hormones are important regulators of metabolism, the concentration of indole-3-acetic acid (IAA), which plays a critical role in regulating many aspects of plant growth and development, was measured in young buds at different developmental stages (Figure 8). The IAA concentration of MS buds was consistently and significantly lower than that of MF buds from sporogenous cell formation to microspore stages, especially during the pollen mother cell formation stage. In addition, considering that several auxin response factor genes were targeted by the differentially-expressed miR160a (Table 2), it is supposed that miRNA160a might play a vital role in the regulation of the IAA signal pathway.

## 3. Discussion

In order to identify and characterize the key miRNAs involved in the regulatory network underlying the abortion process of a novel male sterile mutant line Yu98-8A, high-throughput sequencing was combined with degradome sequencing, quantitative RT-PCR and histological and biochemical analyses. Based on the these analyses, 1588 and 1536 conserved miRNAs, 206 and 205 miRNA-5ps, 165 and 167 miRNA-5ps, 1987 and 1842 hairpins and 347 and 351 novel miRNAs were identified from the MS and MF libraries, respectively. Bioinformatics prediction and analysis showed 100 and 281 different targets of the differentially-expressed miRNAs, including 13 (26.5%, 49) known and 30 (58.9%, 51) novel miRNAs mainly involved in ascorbate and aldarate metabolism, plant hormone signal transduction, metabolic pathways and starch and sucrose metabolism, which indicated the vital and complex roles of miRNAs during the process of male sterility of Yu98-8A.

Target prediction demonstrated that eight of 53 targets of miRNA5658 were predicted, and all eight targets were involved in phytohormonal metabolism and signaling pathways. For example, the auxin-responsive SAUR protein (Cotton_D_gene_10015913), as the largest family of early auxin response factors, plays a pivotal role in cell enlargement [28,29]. BAK1 (Cotton_D_gene_10003171), BKI1 (Cotton_D_gene_10027756 and Cotton_D_gene_10027686) and BRI1 (Cotton_D_gene_10012266) were also degraded by miRNA5658, and BAK1 not only regulates brassinosteroid perception, but also activates BRI1, both of which together mediate cell division, anther development and the brassinosteroid signaling pathway [30,31,32]. The other targets, including Cotton_D_gene_10033147, Cotton_D_gene_10015920 and Cotton_D_gene_10018858, are involved in the salicylic acid, jasmonic acid and abscisic acid signaling pathways. Gibberellic acid signaling promotes the growth and development of plants by degrading DELLA proteins, some of which are major GA repressors during vegetative growth and floral induction [33]. As anticipated, miR473 was not expressed in MF buds in the present study, which targets a DELLA protein-coding gene (Cotton_D_gene_10021396) (Table S6).

Several known miRNAs, such as miRNA156/7 [13], miRNA159 [15], miRNA160 [17], miRNA167 [18,19], miRNA172 [7,12], miRNA319 [34,35,36] and OsmiRNA396d [14], playing important regulatory roles in plants, were also identified in this study. Interestingly, with the exception of miRNA160 and miRNA319, all of the other miRNAs did not show significant differential expression patterns between the MS and MF young buds from the small RNA sequencing data.

A total of eight auxin response factor (ARF) targets of miRNA160a were identified by degradome analysis, and IAA levels in MS buds were consistently and significantly lower than in MF buds from the sporogenous cell formation to microspore development stages (Table 2 and Figure 6), which indicated ARFs targeted by miR160 mainly play important roles in floral development [17,37]. In addition, two TCP family transcription factors (Cotton_D_gene_10039906 and Cotton_D_gene_10003308) of miRNA319a (novel miRNA104) were also identified by both bioinformatics and degradome analysis. It has been well illustrated that all of these transcription factors are involved in the regulation of bud growth, cell proliferation, flower induction and stamen development [34,35,38]. Both miRNA159 and miRNA172 regulate the floral organ development by regulating the expression of their transcription factor targets [7,12,15]. For example, upregulation of miRNA159 expression could result in delayed flowering and disordered anther development in *Arabidopsis* [39].

F-box proteins usually degrade DELLA proteins through a SCF complex during the GA signaling pathway [40]. In the current study, a particular miRNA394a, which targets a specific f-box family protein coding gene (Cotton_D_gene_10011419), was expressed at higher levels in MF buds than that in MS buds. According to Table S5, there was no expression of miRNA473, which targets DELLA protein (Cotton_D_gene_10021396) in MS buds. In addition, GA levels in MS young buds were significantly lower than those of young MF buds [41]. Therefore, it can be speculated that the miRNA cleaves some of the f-box protein-coding genes by miR394a and that no efficient cleavage of DELLA mRNA is present due to significantly weaker expressions of miR394a during the development of buds from the sporogenous cell formation to the meiotic stages. This might be one of the reasons for male abortion in Yu98-8A through blocking GA signal transduction pathways.

miRNA2118a was specifically expressed in MF buds, and 11 of the total targets were pre-mRNA-processing factors and DNA-directed RNA polymerases I, II and III (Table 2 and Table S5). Some of the targets or their paralogs may be involved in miRNA biogenesis. For example, STA1 is an *Arabidopsis* pre-mRNA processing factor that is involved in miRNA biogenesis [42]. In this study, several RNA polymerase III-coding genes were targeted by miRNA2118a (Table 2). It is well known that most plant and animal miRNAs are transcribed by DNA-directed RNA polymerase II and III (Pol II and Pol III) [22,43]. Therefore, these results clearly indicate that miRNAs play several direct or indirect roles contributing to male sterility in Yu98-8A.

## 4. Materials and Methods

### 4.1. Plant Materials

Plants of genic MS line Yu98-8A and the wild-type of Yu98-8A were grown in the field of the Henan Academy of Agricultural Science Xinxiang in Henan province, China. In order to study the relationship between the diameter of the flower bud and pollen stages, buds (<9 mm) were harvested from 10 sterile and 10 fertile plants for cytological and microscopic analysis [41]. Based on microscopic evaluation, the buds from the pollen mother cell formation to meiosis stages were collected from 56 (MS) and 58 (MF) plants from three biological replicates of each line, respectively, snap-frozen in liquid nitrogen and stored at −80 °C for future use.

### 4.2. Histological Analysis

After the sterile and fertile floral buds were fixed, specimen were dehydrated in an ethanol series and embedded in paraffin. Samples were sectioned into ~8-μm sections using a microtome (Leica RM2245, Microsystems, Wetzlar, Germany), mounted on glass sliders and then stained with 0.1% toluidine blue O for 30–60 seconds at room temperature for viewing (Leica DM2500 M, Microsystems) [41].

### 4.3. Extraction, Library Construction and Sequencing of Small RNAs

Total RNA was extracted from equally-mixed young buds of each line using the pBIOZOL Total RNA Extraction Reagent (BioFlux, Fluxion Biosciences Inc., South San Francisco, CA, USA), precipitated with ethanol, dissolved in diethylpyrocarbonate (DEPC) treated water and stored at −80 °C. All RNA samples were examined for protein contamination (A260/A280 ratios) and reagent contamination (A260/A230 ratios) using a NanoDrop ND 1000 spectrophotometer (NanoDrop, Wilmington, DE, USA). The extracted total RNA was then electrophoresized through denatured 15% polyacrylamide gels. Gel fragments with a size range of 18–30 nt were excised, and the small RNA fragments were eluted overnight with 0.5 M NaCl at 4 °C. Fragments were precipitated with 1 mL ethyl ethanol, resuspended in buffer; 5′ and 3′ RNA adapters were added prior to ligation with T4 RNA ligase by the Illumina TruSeq Small RNA Preparation Kit (LC Sciences, Hangzhou, China). The adapter-ligated small RNAs were subsequently transcribed into cDNA by SuperScript^®^ II Reverse Transcriptase (Invitrogen, Carlsbad, CA, USA) and PCR amplified, using primers that were annealed to the ends of the adapters. The amplified cDNA products were purified, recovered and sequenced by Solexa sequencing (BGI, Shenzhen, China).

### 4.4. Computational Analysis of Sequencing Data and Identification of Known Conserved and Novel microRNAs

After the raw sequence reads were extracted, reads of low quality, adapter and insert contaminants, RNAs shorter than 18 nucleotides and polyA were all filtered. The remaining sequences were blasted to the Rfam database (Version:10.1, https://en.wikipedia.org/wiki/Rfam), NCBI GenBank database and repeat-Repbase for ribosomal RNA (rRNA), transfer RNA (tRNA), small nuclear RNA (snRNA), small nucleolar RNA (snoRNA) and other non-coding RNAs, repeat sequences and mRNAs, and all matched sequences were again filtered. The sequences that mapped to TIGR Cotton Transcript Assemblies (http://plantta.jcvi.org/) were then annotated using the Short Oligonucleotide Analysis Package (http://soap.genomics.org.cn), and the unmatched sequences were filtered [23]. Finally, the remaining sequences were mapped to all known plant miRNA sequences to identify the conserved miRNAs by miRBase database (version 19.0, http://www.mirbase.org/), and the perfectly-matched sequences were considered conserved miRNAs. To identify potentially novel miRNAs, cotton transcript assemblies (http://occams.dfci.harvard.edu/pub/bio/tgi/data/Gossypium) from the Dana Farber Cancer Institute were chosen to map unique small RNA sequences and the flanking genome sequence of unique small RNAs using MicroRNA Discovery By Deep Sequencing (MIREAP) (http://sourceforge.net/projects/mireap/). Any potential miRNA must meet some criteria. The first criterion is both the candidate miRNA sequences and their corresponding reverse candidate miRNA* sequences must be detected using high-throughput sequencing. Second, the precursor of any candidate miRNAs should fold secondary hairpin structures; the miRNA and miRNA* sequences must be found on the stem of the precursor; the number of mismatched bases between them must be less than three; and the mature miRNA strand and its complementary miRNA* complex should present two-nucleotide 3' overhangs. Third, within the miRNA/miRNA* duplex, the number of asymmetric bulges must be one or fewer, and the number of bases in the asymmetric bulges must be less than two [44]. Fourth, the potential miRNA precursor must have higher negative minimal folding energy (MFE) and minimal folding energy indexes (MFEI), with the MFEI > 0.8 [45]. The last criterion is the number of mature miRNAs with predicted hairpin, which should not be less than five in the alignment result.

For miRNA differential expression analysis, *p*-values were calculated according to the method by Audic and Claverie, 1997 [46]. Normalized read counts were calculated according to the formula, Normalized read count = (actual miRNA count/total count of clean reads) × 1,000,000. If one miRNA had no read in a library, the normalized read count of this miRNA in the library was arbitrarily set at 0.001 for further calculations. Finally, log_2_ (normalized count of miRNA in MS bud/normalized count) values was used to generate the scatter plots.

### 4.5. Target Prediction and Identification of miRNA by Informatics and Degradome Sequencing

For analyzing the functions of the conserved and novel miRNAs identified in the young buds from the mother pollen formation to meiotic stages, target genes were predicted and identified by bioinformatics and degradome sequencing. Bioinformatic prediction was conducted according to the method by Li et al., 2008 [23]. The main criteria were as follows: first, there were no more than four mismatches between sRNA and target, and no more than two adjacent mismatches in the miRNA/target duplex, no adjacent mismatches in 2–12 bases of the miRNA (5′ end)/target duplex, no mismatches in 10–11 bases of the duplex and no more than 2.5 mismatches, G–U pair counted as 0.5 mismatches, in 1–12 bases of the miRNA (5′ end)/target duplex. Furthermore, the minimum free energy (MFE) of the duplex should be ≥75% of the MFE of the miRNA bound to its perfect complement. Two degradome cDNA libraries were constructed from the young buds of male sterile (MS) and male fertile (MF) plants based on the method by Addo-Quaye et al., 2008; German et al., 2008 [47,48]. Poly (A)-enriched RNAs were ligated to 5′-RNA adapters using T4 RNA ligase (Takara, Dalian, China), and consequently, reverse transcription was performed to generate first-strand cDNAs using an oligo dT primer and SuperScript II RT (Invitrogen, Carlsbad, CA, USA), followed by PCR amplification for six cycles using Phusion Taq and enzyme *Mme* I digestion (NEB, Beijing, China). The digested products were later ligated with double-stranded DNA adapters using T4 DNA ligase, followed by purification by electrophoresis through a 10% PAGE-gel and a PCR amplification for 20 cycles. Finally, the PCR products were purified and used for high-throughput sequencing using Illumina HiSeq 2000 (San Diego, CA, USA). After low quality sequences and adapters were removed, the remaining unique sequence signatures were aligned to the database of cotton transcript assemblies in the Cotton Gene Index (Release 11.0) using SOAP software. Identification and classification of categories of the miRNA targets were then conducted using a public software package, CleaveLand 3.0 [49]. Finally, gene ontology (GO) (http://www.geneontology.org/) was used for the assignment of the identified target genes according to the previous described method [50].

To assign putative functions to assembled unigenes, a set of sequential BLAST was searched against sequences in NCBI [51,52], and the Kyoto Encyclopedia Gene and Genomes (KEGG) database (http://www.genome.jp/kegg/ko.html) was used to identify significantly-enriched metabolic pathways of targets of differentially-expressed miRNAs [53].

### 4.6. Expression Analysis of miRNAs by RT-qPCR

RT-qPCR was carried out to estimate the expression differences of miRNAs and their corresponding target genes between young MS and MF buds. All primers were designed according to corresponding gene sequences with Primer Premier 5.0 (Premier, Toronto, ON, Canada) (Table S8). The RT-qPCR assay using 2 μL (5 ng/μL) cDNA and SYBR Green PCR Master Mix (Takara) was performed in triplicate on an ABI Prism 7000 Real-time PCR system (Foster City, CA, USA). Twenty-microliter RT-qPCR reactions were incubated in a 96-well plate at 95 °C for 10 min, followed by 40 cycles of 95 °C for 15 s and 60 °C for 60 s. The cotton endogenous 5S rRNA gene was used to normalize the amount of gene-specific RT-PCR products [54], and the relative expression of genes was quantified using the 2^−ΔΔ*C*t^ method [55].

### 4.7. Measurement of the Indole-3 Acetic Acid Content of Young Buds

The extraction and purification of endogenous phytohormones prior to the immunoassay were carried out by the enzyme-linked immunosorbent assay (ELISA) according to the methods by Yang et al., 2001, and Cui et al., 2005 [56,57], with minor modifications. Briefly, 0.5 g of lyophilized ground buds were homogenized through repeated inversion in pre-chilled 80% aqueous methanol containing 1 mM butylated hydroxytoluene. The supernatant was collected, centrifuged at 5000× *g* for 20 min at 4 °C, and the precipitate was re-extracted and re-centrifuged. The crude extracts were passed through a Sep-Pak C18 cartridge (Waters, Milford, MA, USA), prewashed with 10 mL 100% (*w*/*v*) and 5 mL 80% (*v*/*v*) methanol, respectively. Then, the filtrates eluted with 10 mL 100% (*v*/*v*) methanol and 10 mL ether from the columns were dried in N_2_ gas, and the resultant residues were dissolved in phosphate-buffered saline (PBS, 0.01M, pH 7.4). The levels of indole-3-acetic acid (IAA) were then determined using monoclonal antibodies (Affandi, Shanghai, China) and determined as pg per gram fresh weight of sample. Absorbance of the developed color was measured at 490 nm using a microplate reader (M-SPmax250, Wako Pure Chem, Tokyo, Japan) [58].

### 4.8. Statistical Analysis

The analysis of variance (ANOVA) was performed on phytohormonal content, and enzymatic activity and all significant differences were examined according to Tukey’s test using DPS 6.05 software at *p* < 0.05 [59].

## 5. Conclusions

Based on the use of microscopic observation to identify floral developmental stages, 1588 and 1536 conserved miRNAs and 347 and 351 novel miRNAs were further identified and predicted during the pollen abortion stages from MS (male sterile) and MF (male fertile) young buds of cotton plants. Two independent small RNA libraries were constructed and sequenced from the young buds collected from the sporogenous cell formation to meiosis stage of the male sterile line Yu98-8A and the near-isogenic wild-type. miRNA expression profiles revealed that 49 conserved and 51 novel miRNAs were differentially expressed. Target analysis based on bioinformatics and degradomes simultaneously indicated the regulatory complexity of miRNAs during flower induction and development. Further, RT-qPCR and physiological analysis indicated that, among the different KEGG pathways, the indole-3-acetic acid (IAA) and gibberellic acetic acid (GA) signaling transduction pathways may play pivotal regulatory functions in male abortion.

## Figures and Tables

**Table 1:** Small RNA sequences from two flower bud libraries.

Type	Library of MS Buds		Library of MF Buds	
	Unique sRNAs (%)	Total sRNAs (%)	Unique sRNAs (%)	Total sRNAs (%)
Total	5,410,937 (100)	11,002,282 (100)	5,151,550 (100)	10,663,659 (100)
Exon_antisense	49,962 (0.92)	99,307 (0.9)	44,463 (0.86)	91,843 (0.86)
Exon_sense	70,831 (1.31)	120,224 (1.09)	6,5017 (1.26)	110,560 (1.04)
Intron_antisense	44,021 (0.81)	116,204 (1.06)	42,055 (0.82)	114,356 (1.07)
Intron_sense	54,670 (1.01)	136,727 (1.24)	52,371 (1.02)	133,319 (1.25)
miRNA	15,511 (0.29)	763,877 (6.94)	15,476 (0.3)	718,317 (6.74)
rRNA	28,523 (0.53)	179,086 (1.63)	28,326 (0.55)	189,958 (1.78)
repeat	227,017 (4.2)	635,535 (5.78)	213,793 (4.15)	598,611 (5.61)
snRNA	2119 (0.04)	5134 (0.05)	1801 (0.03)	4478 (0.04)
snoRNA	850 (0.02)	2271 (0.02)	691 (0.01)	2441 (0.02)
tRNA	6206 (0.11)	61,552 (0.56)	4,459 (0.09)	46,324 (0.43)
Unannotated	4,911,227 (90.76)	8,882,365 (80.73)	4,683,098 (90.91)	8,653,452 (81.15)

**Table 2:** Targets of differentially-expressed miRNAs identified by degradome analysis.

miRNA	Target	
	Gene ID	Function Annotation
miR160a	Cotton_D_gene_10025306	auxin response factor 18-like
	Cotton_D_gene_10006397	putative auxin response factor ARF16
	Cotton_D_gene_10005922	auxin response factor 18-like
	Cotton_D_gene_10019554	auxin response factor
	Cotton_D_gene_10010678	putative auxin response factor
	Cotton_D_gene_10008456	auxin response factor
	Cotton_D_gene_10002224	auxin response factor
miR2118a_3p	Cotton_D_gene_10027482	DNA-directed RNA polymerases I, II, and III subunit RPABC1
	Cotton_D_gene_10027759	phospholipase D
	Cotton_D_gene_10025494	pre-mRNA-processing factor 40
	Cotton_D_gene_10027502	DNA-directed RNA polymerases I, II, and III subunit RPABC1
	Cotton_D_gene_10027449	DNA-directed RNA polymerases I, II, and III subunit RPABC1
	Cotton_D_gene_10027508	maintenance of ploidy protein MOB1
	Cotton_D_gene_10027458	NBS-LRR resistance protein-like protein
	Cotton_D_gene_10027448	TIR-NBS-LRR resistance protein
	Cotton_D_gene_10027489	DNA-directed RNA polymerases I, II, and III subunit RPABC1
	Cotton_D_gene_10039426	nitrate reductase (NADH)
	Cotton_D_gene_10017763	uncharacterized protein
	Cotton_D_gene_10027457	DNA-directed RNA polymerases I, II, and III subunit RPABC1
	Cotton_D_gene_10027494	DNA-directed RNA polymerases I, II, and III subunit RPABC1
	Cotton_D_gene_10017214	uncharacterized protein
	Cotton_D_gene_10012644	DNA-directed RNA polymerases I, II, and III subunit RPABC1
	Cotton_D_gene_10031090	protein phosphatase 1, catalytic subunit
	Cotton_D_gene_10027490	ATP binding protein
	Cotton_D_gene_10027493	DNA-directed RNA polymerases I, II, and III subunit RPABC1
	Cotton_D_gene_10027495	maintenance of ploidy protein MOB1
	Cotton_D_gene_10027490	ATP binding protein
	Cotton_D_gene_10027485	DNA-directed RNA polymerases I, II, and III subunit RPABC1
	Cotton_D_gene_10027456	NBS-LRR resistance protein-like protein
	Cotton_D_gene_10027452	TIR-NBS-LRR resistance protein
	Cotton_D_gene_10027491	hypothetical protein
	Cotton_D_gene_10027497	DNA-directed RNA polymerases I, II, and III subunit RPABC1
	Cotton_D_gene_10027484	hypothetical protein
miR394a	Cotton_D_gene_10011419	F-box family protein
miR397a	Cotton_D_gene_10033270	laccase 1b
miR5224a	Cotton_D_gene_10020595	cysteine-rich receptor-like protein kinase 25-like
novel_mir_104	Cotton_D_gene_10039906	TCP4
	Cotton_D_gene_10003308	TCP4
novel_mir_168	Cotton_D_gene_10018721	predicted protein
novel_mir_20	Cotton_D_gene_10009655	chromatin licensing and DNA replication factor 1

Only the targets with a *p*-value of the cleave site of <0.05 are listed.

## Supplementary Materials

Supplementary materials can be found at www.mdpi.com/1422-0067/17/10/1677/s1.

## References

[1] Sunilkumar G., Campbell L.M., Puckhaber L., Stipanovic R.D., Rathore K.S. (2006). Engineering cottonseed for use in human nutrition by tissue-specific reduction of toxic gossypol. Proc. Natl. Acad. Sci. USA.

[2] Jones-Rhoades M.W., Bartel D.P., Bartel B. (2006). MicroRNAs and their regulatory roles in plants. Annu. Rev. Plant Biol..

[3] Wu L., Zhou H., Zhang Q., Zhang J., Ni F., Liu C., Qi Y. (2010). DNA methylation mediated by a microRNA pathway. Mol. Cell.

[4] Reyes J.L., Chua N.H. (2007). ABA induction of miR159 controls transcript levels of two MYB factors during Arabidopsis seed germination. Plant J..

[5] Gutierrez L., Bussell J.D., Pacurar D.I., Schwambach J., Pacurar M., Bellini C. (2009). Phenotypic plasticity of adventitious rooting in Arabidopsis is controlled by complex regulation of AUXIN RESPONSE FACTOR transcripts and microRNA abundance. Plant Cell.

[6] Floyd S.K., Bowman J.L. (2004). Gene regulation: Ancient microRNA target sequences in plants. Nature.

[7] Chen X. (2004). A microRNA as a translational repressor of APETALA2 in *Arabidopsis* flower development. Science.

[8] Millar A.A., Gubler F. (2005). The Arabidopsis GAMYB-like genes, MYB33 and MYB65, are microRNA-regulated genes that redundantly facilitate anther development. Plant Cell.

[9] Wang T.Z., Chen L., Zhao M.G., Tian Q.Y., Zhang W.H. (2011). Identification of drought-responsive microRNAs and their targets in *Medicago truncatula* by genome-wide high-throughput sequencing and degradome analysis. BMC Genom..

[10] Ding D., Zhang L., Wang H., Liu Z., Zhang Z., Zheng Y. (2009). Differential expression of miRNAs in response to salt stress in maize roots. Ann. Bot..

[11] Yin Z.J., Li Y., Han X.L., Shen F.F. (2012). Genome-wide profiling of miRNAs and other small non-coding RNAs in the *Verticillium dahliae*-inoculated Cotton Roots. PLoS ONE.

[12] Aukerman M.J., Sakai H. (2003). Regulation of flowering time and floral organ identity by a microRNA and its APETALA2 -like target genes. Plant Cell.

[13] Xing S.P., Salinas M., Höhmann S., Berndtgen R., Huijser P. (2010). MiR156-targeted and nontargeted SBP-box transcription factors act in concert to secure male fertility in *Arabidopsis*. Plant Cell.

[14] Liu H.H., Guo S.Y., Xu Y.Y., Li H., Zhang Z.Y., Zhang J., Xu S.J., Zhang C., Chong K. (2014). OsmiR396d-regulated OsGRFs function in floral organogenesis in rice through binding to their targets OsJMJ706 and OsCR4. Plant Physiol..

[15] Tsuji H., Aya K., Ueguchi-Tanaka M., Shimada Y., Nakazono M., Watanabe R., Nishizawa N.K., Gomi K., Shimada A., Kitano H. (2006). GAMYB controls different sets of genes and is differentially regulated by microRNA in aleurone cells and anthers. Plant J..

[16] Zheng B.L., Chen X.M., McCormick S. (2011). The anaphase-promoting complex is a dual integrator that regulates both microRNA-mediated transcriptional regulation of cyclin B1 and degradation of cyclin B1 during *Arabidopsis* male gametophyte development. Plant Cell.

[17] Mallory A.C., Bartel D.P., Bartel B. (2005). MicroRNA-directed regulation of *Arabidopsis* AUXIN RESPONSE FACTOR17 is essential for proper development and modulates expression of early auxin response genes. Plant Cell.

[18] Nagpal P., Ellis C.M., Weber H., Ploense S.E., Barkawi L.S., Guilfoyle T.J., Hagen G., Alonso J.M., Cohen J.D., Farmer E.E. (2005). Auxin response factors ARF6 and ARF8 promote jasmonic acid production and flower maturation. Development.

[19] Wu M.F., Tian Q., Reed J.W. (2006). *Arabidopsis* microRNA167 controls patterns of ARF6 and ARF8 expression, and regulates both female and male reproduction. Development.

[20] Shen Y.O., Zhang Z.M., Lin H.J., Liu H.L., Chen J., Peng H., Cao M.J., Rong T.Z., Pan G.T. (2011). Cytoplasmic male sterility-regulated novel microRNAs from maize. Funct. Integr. Genom..

[21] Yang J.J., Zhang H.Y., Zheng Y.Z., Ding Y. (2015). Comparative expression profiling of miRNAs between the cytoplasmic male sterile line MeixiangA and its maintainer line MeixiangB during rice anther development. Planta.

[22] Yang J.H., Liu X.Y., Xu B.C., Zhao N., Yang X.D., Zhang M.F. (2013). Identification of miRNAs and their targets using high-throughput sequencing and degradome analysis in cytoplasmic male-sterile and its maintainer fertile lines of *Brassica juncea*. BMC Genom..

[23] Wei M.M., Wei H.L., Wu M., Song M.Z., Zhang J.F., Yu J.W., Fan S.L., Yu S.X. (2013). Comparative expression profiling of miRNA during anther development in genetic male sterile and wild type cotton. BMC Plant Biol..

[24] Jiang J.X., Jiang J.J., Yang Y.F., Cao J.S. (2013). Identification of microRNAs potentially involved in male sterility of *Brassica campestris ssp. chinensis* using microRNA array and quantitative RT-PCR assays. Cell Mol. Biol. Lett..

[25] Jiang J.X., Lv M.L., Liang Y., Ma Z.M., Cao J.S. (2014). Identification of novel and conserved miRNAs involved in pollen development in *Brassica campestris* ssp. *chinensis* by high-throughput sequencing and degradome analysis. BMC Genom..

[26] Zhang W., Xie Y., Xu L., Wang Y., Zhu X.W., Wang R.H., Zhang Y., Muleke E.M., Liu L.W. (2016). Identification of microRNAs and their target genes explores miRNA-mediated regulatory network of cytoplasmic male sterility occurrence during anther development in Radish (*Raphanus sativus* L.). Front. Plant Sci..

[27] Ding X.L., Li J.J., Zhang H., He T.T., Han S.H., Li Y.W., Yang S.P., Gai J.Y. (2016). Identification of miRNAs and their targets by high-throughput sequencing and degradome analysis in cytoplasmic male-sterile line NJCMS1A and its maintainer NJCMS1B of soybean. BMC Genom..

[28] Markakis M.N., Boron A.K., Van Loock B., Saini K., Cirera S., Verbelen J.P., Vissenberg K. (2013). Characterization of a small auxin-up RNA (SAUR)-like gene involved in *Arabidopsis thaliana* development. PLoS ONE.

[29] Ren H., Gray W.M. (2015). SAUR Proteins as effectors of hormonal and environmental signals in plant growth. Mol. Plant.

[30] He K., Xu S.B., Li J. (2013). BAK1 directly regulates brassinosteroid perception and BRI1 activation. J. Integr. Plant Biol..

[31] Li J. (2010). Multi-tasking of somatic embryogenesis receptor-like protein kinases. Curr. Opin. Plant Biol..

[32] Zhu J.Y., Sae-Seaw J., Wang Z.Y. (2013). Brassinosteroid signalling. Development.

[33] Mutasa-Göttgens E., Hedden P. (2009). Gibberellin as a factor in floral regulatory networks. J. Exp. Bot..

[34] Nag A., King S., Jack T. (2009). miR319a targeting of TCP4 is critical for petal growth and development in Arabidopsis. Proc. Natl. Acad. Sci. USA.

[35] Rubio-Somoza I., Weigel D. (2013). Coordination of flower maturation by a regulatory circuit of three microRNAs. PLoS Genet..

[36] Wang H., Mao Y.F., Yang J., He Y.K. (2015). TCP 24 modulates secondary cell wall thickening and anther endothecium and anther development. Front. Plant Sci..

[37] Liu X., Huang J., Wang Y., Khanna K., Xie Z., Owen H.A., Zhao D. (2010). The role of floral organs in carpels, an *Arabdopsis* loss-of-function mutation in microRNA160a, in organogenesis and the mechanism regulating its expression. Plant J..

[38] Xing L.B., Zhang D., Zhao C.P., Li Y.M., Ma J.J., An N., Han M.Y. (2016). Shoot bending promotes flower bud formation by miRNA-mediated regulation in apple (*Malus domestica Borkh*.). Plant Biotechnol. J..

[39] Achard P., Herr A., Baulcombe D.C., Harberd N.P. (2004). Modulation of floral development by a gibberellin-regulated microRNA. Development.

[40] Ariizumi T., Lawrence P.K., Steber C.M. (2011). The role of two f-box proteins, SLEEPY1 and SNEEZY, in Arabidopsis gibberellin signaling. Plant Physiol..

[41] Fang W.P., Zhao F.A., Sun Y., Xie D.Y., Sun L., Xu Z.Z., Zhu W., Yang L.R., Zhao Y.M., Lv S.P. (2015). Transcriptomic profiling reveals complex molecular regulation in cotton genic male sterile mutant Yu98-8A. PLoS ONE.

[42] Chaabane S.B., Liu R.Y., Chinnusamy V., Kwon Y., Park J.H., Kim S.Y., Zhu J.K., Yang S.W., Lee B.H. (2013). STA1, an *Arabidopsis* pre-mRNA processing factor 6 homolog, is a new player involved in miRNA biogenesis. Nucleic Acids Res..

[43] Lee Y., Kim M., Han J., Yeom K.H., Lee S., Baek S.H., Kim V.N. (2004). microRNA genes are transcribed by RNA polymerase II. EMBO J..

[44] Meyers B.C., Axtell M.J., Bartel B., Bartel D.P., Baulcombe D., Bowman J.L., Cao X., Carrington J.C., Chen X., Green P.J. (2008). Criteria for annotation of plant MicroRNAs. Plant Cell.

[45] Zhang B.H., Pan X.P., Cox S.B., Cobb G.P., Anderson T.A. (2006). Evidence that miRNAs are different from other RNAs. Cell Mol. Life Sci..

[46] Audic S., Claverie J.M. (1997). The significance of digital gene expression profiles. Genome Res..

[47] Addo-Quaye C., Eshoo T.W., Bartel D.P., Axtell M.J. (2008). Endogenous siRNA and miRNA targets identified by sequencing of the *Arabidopsis* degradome. Curr. Biol..

[48] German M.A., Pillay M., Jeong D.H., Hetawal A., Luo S., Janardhanan P., Kannan V., Rymarquis L.A., Nobuta K., German R. (2008). Global identification of microRNA-target RNA pairs by parallel analysis of RNA ends. Nat. Biotechnol..

[49] Addo-Quaye C., Miller W., Axtell M.J. (2009). CleaveLand: A pipeline for using degradome data to find cleaved small RNA targets. Bioinformatics.

[50] Du Z., Zhou X., Ling Y., Zhang Z.H., Su Z. (2010). AgriGO: A GO analysis tool kit for the agricultural community. Nucleic Acids Res..

[51] Altschul S.F., Madden T.L., Schäffer A.A., Zhang J., Zhang Z., Miller W., Lipman D.J. (1997). Gapped BLAST and PSI-BLAST: A new generation of protein database search programs. Nucleic Acids Res..

[52] Conesa A., Götz S., García-Gómez J.M., Terol J., Talón M., Robles M. (2005). Blast2GO: A universal tool for annotation, visualization and analysis in functional genomics research. Bioinformatics.

[53] Kanehisa M., Araki M., Goto S., Hattori M., Hirakawa M., Itoh M., Katayama T., Kawashima S., Okuda S., Tokimatsu T. (2008). KEGG for linking genomes to life and the environment. Nucleic Acids Res..

[54] Ma C., Lu Y., Bai S.L., Zhang W.N., Duan X.W., Meng D., Wang Z.G., Wang A.D., Zhou Z.S., Li T.Z. (2014). Cloning and characterization of miRNAs and their targets, including a novel miRNA-targeted NBS-LRR protein class gene in apple (Golden Delicious). Mol. Plant.

[55] Livak K.J., Schmittgen T.D. (2001). Analysis of relative gene expression data using real-time quantitative PCR and the 2^−ΔΔ*C*t^ Method. Methods.

[56] Yang J.C., Zhang J.H., Wang Z.Q., Zhu Q.S., Wang W. (2001). Hormonal changes in the grains of rice subjected to water stress during grain filling. Plant Physiol..

[57] Cui D., Neill S.J., Tang Z., Cai W. (2005). Gibberellin-regulated XET is differentially induced by auxin in rice leaf sheath bases during gravitropic bending. J. Exp. Bot..

[58] Fang W.P., Xie D.Y., Zhu H.Q., Li W., Xu Z.Z., Yang L.R., Li Z.F., Sun L., Wang J.X., Nie L.H. (2015). Comparative proteomic analysis of *Gossypium thurberi* in response to *Verticillium dahliae* inoculation. Int. J. Mol. Sci..

[59] Tang Q.Y., Zhang C.X. (2013). Data Processing System (DPS) software with experimental design, statistical analysis and data mining developed for use in entomological research. Insect Sci..

