# Supplementary material for: Identification and Functional Analysis of microRNAs Involved in the Anther Development in Cotton Genic Male Sterile Line Yu98-8A
Source: Int J Mol Sci. 2016 Oct 7;17(10):1677. doi: 10.3390/ijms17101677 (PMC5085710; doi:10.3390/ijms17101677)
Supplement: Supplementary file 1 [file ijms-17-01677-s001.zip › ijms-142136-supplementary-publish.pdf]

Supplementary Materials: Identification and Functional Analysis of microRNAs Involved in the Anther Development in Cotton Genic Male Sterile Line Yu98-8A

Xiaojie Yang, Yuanming Zhao, Deyi Xie, Yao Sun, Xunlu Zhu, Nardana Esmaeili, Zuoren Yang, Ye Wang, Guo Yin, Shuping Lv, Lihong Nie, Zhongjie Tang, Fu'an Zhao, Wu Li, Neelam Mishra, Li Sun, Wei Zhu and Weiping Fang

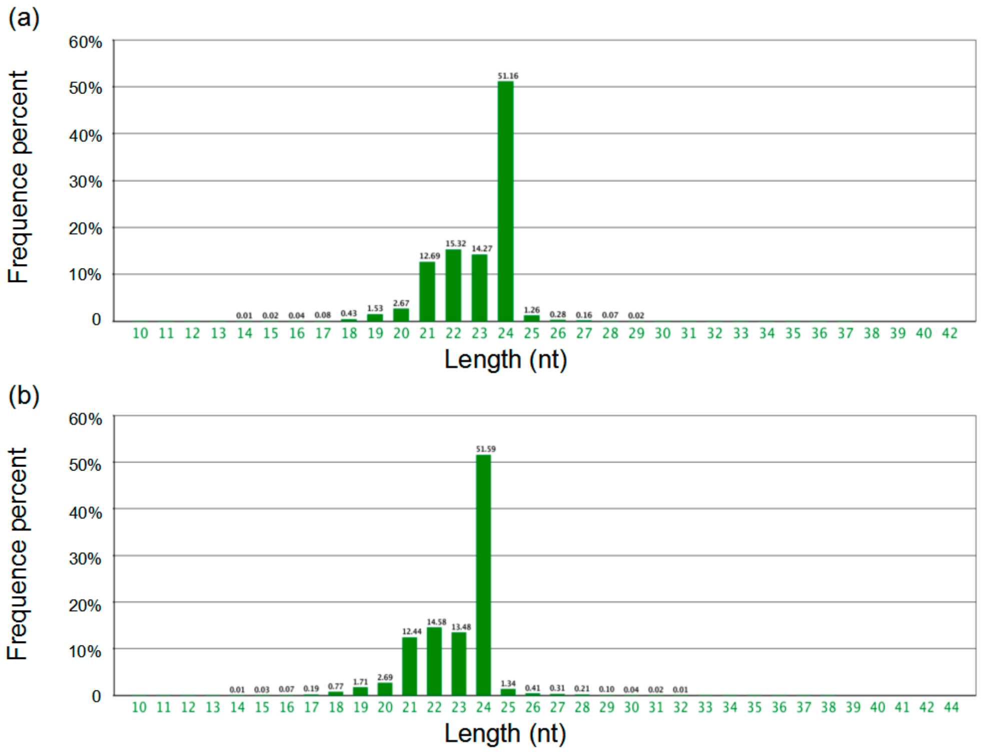

Figure S1. Length distribution of small RNAs.

Table S1. Summary of sequencing data from two individual libraries.

Type	Library of MS Buds		Library of MF Buds	
	Count	Percent (%)	Count	Percent (%)
Total reads	11,095,604	–	10,741,527	–
High_quality	11,044,443	100.00	10,688,607	100.00
3′ adapter_null	2073	0.02	2869	0.03
Insert_null	312	0.00	341	0.00
5′ adapter_null	6529	0.06	5927	0.06
Shorter_than_18nt	32,275	0.29	14,930	0.14
PolyA	972	0.01	881	0.01
Clean_reads	11,002,282	99.62	10,663,659	99.77

Table S2. Statistics of conseved miRNAs from two individual libraries.

Type		No. of miRNA	No. of miRNA-5p	No. of miRNA-3p	No. of Hairpin
No. of miRBase 19		4412	766	762	5127
No. of identified	MS	1588	206	165	1897
	MF	1536	205	167	1842

Table S6. Targets of differentially expressed known and novel miRNAs predicted by informatics.

miRNA	Targets	
	Number	Gene ID
miR160a	8	Cotton_D_gene_10032280, Cotton_D_gene_10025306, Cotton_D_gene_10019554, Cotton_D_gene_10010678, Cotton_D_gene_10008456, Cotton_D_gene_10005922, Cotton_D_gene_10006397, Cotton_D_gene_10002224
miR2111a	1	Cotton_D_gene_10019350
miR2118a-3p	8	Cotton_D_gene_10011549, Cotton_D_gene_10015239, Cotton_D_gene_10003169, Cotton_D_gene_10012641, Cotton_D_gene_10012642, Cotton_D_gene_10012644, Cotton_D_gene_10001596, Cotton_D_gene_10002457
miR2911	1	Cotton_D_gene_10037642
miR319	1	Cotton_D_gene_10015208
miR394a	2	Cotton_D_gene_10024254, Cotton_D_gene_10011419
miR397a	20	Cotton_D_gene_10032147, Cotton_D_gene_10024560, Cotton_D_gene_10024579, Cotton_D_gene_10025270, Cotton_D_gene_10034883, Cotton_D_gene_10033270, Cotton_D_gene_10029334, Cotton_D_gene_10029336, Cotton_D_gene_10036799, Cotton_D_gene_10034274, Cotton_D_gene_10034277, Cotton_D_gene_10034278, Cotton_D_gene_10034326, Cotton_D_gene_10010424, Cotton_D_gene_10024113, Cotton_D_gene_10021697, Cotton_D_gene_10004240, Cotton_D_gene_100042422, Cotton_D_gene_10004255, Cotton_D_gene_10009260
miR473	2	Cotton_D_gene_10021396, Cotton_D_gene_10021796
miR5224a	1	Cotton_D_gene_10013047
miR5338	2	Cotton_D_gene_10025591, Cotton_D_gene_10025592
miR5658	53	Cotton_D_gene_10030644, Cotton_D_gene_10030730, Cotton_D_gene_10032182, Cotton_D_gene_10032194, Cotton_D_gene_10024719, Cotton_D_gene_10025777, Cotton_D_gene_10033147, Cotton_D_gene_10040494, Cotton_D_gene_10025206, Cotton_D_gene_10028544, Cotton_D_gene_10027756, Cotton_D_gene_10037721, Cotton_D_gene_10015441, Cotton_D_gene_10028340, Cotton_D_gene_10029792, Cotton_D_gene_10029828, Cotton_D_gene_10031210, Cotton_D_gene_10028210, Cotton_D_gene_10013894, Cotton_D_gene_10013990, Cotton_D_gene_10012955, Cotton_D_gene_10030124, Cotton_D_gene_10012266, Cotton_D_gene_10012379, Cotton_D_gene_10027686, Cotton_D_gene_10018858, Cotton_D_gene_10016981, Cotton_D_gene_10034540, Cotton_D_gene_10025541, Cotton_D_gene_10010232, Cotton_D_gene_10012128, Cotton_D_gene_10037240, Cotton_D_gene_10037435, Cotton_D_gene_10009431, Cotton_D_gene_10017165, Cotton_D_gene_10015913, Cotton_D_gene_10015920, Cotton_D_gene_10009137, Cotton_D_gene_10006267, Cotton_D_gene_10013688, Cotton_D_gene_10004867, Cotton_D_gene_10008830, Cotton_D_gene_10008830, Cotton_D_gene_10009653, Cotton_D_gene_10011608, Cotton_D_gene_10017453, Cotton_D_gene_10004743, Cotton_D_gene_10008407, Cotton_D_gene_10003171, Cotton_D_gene_10012070, Cotton_D_gene_10002589, Cotton_D_gene_10000877, Cotton_D_gene_10000377
miR5825	1	Cotton_D_gene_10024045
miR6247	1	Cotton_D_gene_10034386
novel_mir_104	3	Cotton_D_gene_10006847, Cotton_D_gene_10003369, Cotton_D_gene_10020936
novel_mir_137	2	Cotton_D_gene_10036714, Cotton_D_gene_10016627
novel_mir_165	2	Cotton_D_gene_10035777, Cotton_D_gene_10001001
novel_mir_168	1	Cotton_D_gene_10024580
novel_mir_20	1	Cotton_D_gene_10035647
novel_mir_204	1	Cotton_D_gene_10034932
novel_mir_206	1	Cotton_D_gene_10014577

Table S6. *Cont.*

miRNA	Targets	
	**Number**	**Gene ID**
novel_mir_22	13	Cotton_D_gene_10040158, Cotton_D_gene_10034716, Cotton_D_gene_10018762, Cotton_D_gene_10028326, Cotton_D_gene_10028249, Cotton_D_gene_10036613, Cotton_D_gene_10020700, Cotton_D_gene_10019674, Cotton_D_gene_10019674, Cotton_D_gene_10021118, Cotton_D_gene_10006199, Cotton_D_gene_10013041, Cotton_D_gene_10010354
novel_mir_229	1	Cotton_D_gene_10011581
novel_mir_23	109	Cotton_D_gene_10040129, Cotton_D_gene_10032171, Cotton_D_gene_10029877, Cotton_D_gene_10034713, Cotton_D_gene_10040827, Cotton_D_gene_10033566, Cotton_D_gene_10024628, Cotton_D_gene_10035245, Cotton_D_gene_10035277, Cotton_D_gene_10035417, Cotton_D_gene_10036178, Cotton_D_gene_10022905, Cotton_D_gene_10022967, Cotton_D_gene_10023060, Cotton_D_gene_10039648, Cotton_D_gene_10026515, Cotton_D_gene_10038967, Cotton_D_gene_10028050, Cotton_D_gene_10038536, Cotton_D_gene_10019695, Cotton_D_gene_10036227, Cotton_D_gene_10036307, Cotton_D_gene_10035007, Cotton_D_gene_10035041, Cotton_D_gene_10027927, Cotton_D_gene_10039707, Cotton_D_gene_10039717, Cotton_D_gene_10039839, Cotton_D_gene_10039851, Cotton_D_gene_10015463, Cotton_D_gene_10038426, Cotton_D_gene_10023706, Cotton_D_gene_10029656, Cotton_D_gene_10026941, Cotton_D_gene_10022078, Cotton_D_gene_10022107, Cotton_D_gene_10027131, Cotton_D_gene_10027231, Cotton_D_gene_10014337, Cotton_D_gene_10014622, Cotton_D_gene_10014664, Cotton_D_gene_10034832, Cotton_D_gene_10028276, Cotton_D_gene_10015648, Cotton_D_gene_10014081, Cotton_D_gene_10025649, Cotton_D_gene_10025697, Cotton_D_gene_10023925, Cotton_D_gene_10016539, Cotton_D_gene_10016543, Cotton_D_gene_10033257, Cotton_D_gene_10036714, Cotton_D_gene_10018882, Cotton_D_gene_10020810, Cotton_D_gene_10023437, Cotton_D_gene_10034402, Cotton_D_gene_10034442, Cotton_D_gene_10034491, Cotton_D_gene_10036442, Cotton_D_gene_10037481, Cotton_D_gene_10024015, Cotton_D_gene_10028955, Cotton_D_gene_10014876, Cotton_D_gene_10027249, Cotton_D_gene_10021781, Cotton_D_gene_10015934, Cotton_D_gene_10010765, Cotton_D_gene_10026366, Cotton_D_gene_10026059, Cotton_D_gene_10019553, Cotton_D_gene_10019572, Cotton_D_gene_10008774, Cotton_D_gene_10008282, Cotton_D_gene_10010670, Cotton_D_gene_10006258, Cotton_D_gene_10007195, Cotton_D_gene_10013740, Cotton_D_gene_10011451, Cotton_D_gene_10005104, Cotton_D_gene_10024309, Cotton_D_gene_10024483, Cotton_D_gene_10024485, Cotton_D_gene_10008894, Cotton_D_gene_10027070, Cotton_D_gene_10006537, Cotton_D_gene_10004797, Cotton_D_gene_10011063, Cotton_D_gene_10005095, Cotton_D_gene_10010752, Cotton_D_gene_10015702, Cotton_D_gene_10013027, Cotton_D_gene_10006381, Cotton_D_gene_10004377, Cotton_D_gene_10006431, Cotton_D_gene_10014130, Cotton_D_gene_10010301, Cotton_D_gene_10010323, Cotton_D_gene_10004130, Cotton_D_gene_10003881, Cotton_D_gene_10002338, Cotton_D_gene_10006202, Cotton_D_gene_10004652, Cotton_D_gene_10002911, Cotton_D_gene_10003495, Cotton_D_gene_10001342, Cotton_D_gene_10001247, Cotton_D_gene_10002984, Cotton_D_gene_10001974, Cotton_D_gene_10000516

Table S6. *Cont.*

miRNA	Targets	
	Number	Gene ID
novel_mir_265	112	Cotton_D_gene_10040068, Cotton_D_gene_10040103, Cotton_D_gene_10038593, Cotton_D_gene_10038598, Cotton_D_gene_10032522, Cotton_D_gene_10034237, Cotton_D_gene_10031989, Cotton_D_gene_10030975, Cotton_D_gene_10029885, Cotton_D_gene_10040808, Cotton_D_gene_10025019, Cotton_D_gene_10039279, Cotton_D_gene_10039375, Cotton_D_gene_10035228, Cotton_D_gene_10023322, Cotton_D_gene_10036135, Cotton_D_gene_10036150, Cotton_D_gene_10025954, Cotton_D_gene_10022902, Cotton_D_gene_10022916, Cotton_D_gene_10039579, Cotton_D_gene_10039034, Cotton_D_gene_10039228, Cotton_D_gene_10028059, Cotton_D_gene_10038949, Cotton_D_gene_10028723, Cotton_D_gene_10028745, Cotton_D_gene_10018238, Cotton_D_gene_10027799, Cotton_D_gene_10036234, Cotton_D_gene_10036906, Cotton_D_gene_10039994, Cotton_D_gene_10028374, Cotton_D_gene_10029057, Cotton_D_gene_10018516, Cotton_D_gene_10027174, Cotton_D_gene_10032484, Cotton_D_gene_10014631, Cotton_D_gene_10026671, Cotton_D_gene_10024224, Cotton_D_gene_10028223, Cotton_D_gene_10031618, Cotton_D_gene_10018624, Cotton_D_gene_10022651, Cotton_D_gene_10025667, Cotton_D_gene_10025671, Cotton_D_gene_10030045, Cotton_D_gene_10030151, Cotton_D_gene_10030155, Cotton_D_gene_10030168, Cotton_D_gene_10016547, Cotton_D_gene_10027631, Cotton_D_gene_10029463, Cotton_D_gene_10029500, Cotton_D_gene_10022547, Cotton_D_gene_10014388, Cotton_D_gene_10014984, Cotton_D_gene_10036433, Cotton_D_gene_10012163, Cotton_D_gene_10023836, Cotton_D_gene_10014246, Cotton_D_gene_10026232, Cotton_D_gene_10020591, Cotton_D_gene_10027255, Cotton_D_gene_10017644, Cotton_D_gene_10015894, Cotton_D_gene_10015967, Cotton_D_gene_10017709, Cotton_D_gene_10013332, Cotton_D_gene_10010791, Cotton_D_gene_10026326, Cotton_D_gene_10019565, Cotton_D_gene_10019671, Cotton_D_gene_10014452, Cotton_D_gene_10009731, Cotton_D_gene_10010653, Cotton_D_gene_10022158, Cotton_D_gene_10008693, Cotton_D_gene_10007237, Cotton_D_gene_10014793, Cotton_D_gene_10009911, Cotton_D_gene_10024256, Cotton_D_gene_10024260, Cotton_D_gene_10024334, Cotton_D_gene_10006467, Cotton_D_gene_10005490, Cotton_D_gene_10027017, Cotton_D_gene_10011070, Cotton_D_gene_10007253, Cotton_D_gene_10004512, Cotton_D_gene_10007516, Cotton_D_gene_10017424, Cotton_D_gene_10015710, Cotton_D_gene_10003386, Cotton_D_gene_10014150, Cotton_D_gene_10020994, Cotton_D_gene_10003585, Cotton_D_gene_10012054, Cotton_D_gene_10003460, Cotton_D_gene_10008234, Cotton_D_gene_10003065, Cotton_D_gene_10010271, Cotton_D_gene_10002056, Cotton_D_gene_10001890, Cotton_D_gene_10001787, Cotton_D_gene_10001634, Cotton_D_gene_10001041, Cotton_D_gene_10001000, Cotton_D_gene_10002545, Cotton_D_gene_10000403, Cotton_D_gene_10000672, Cotton_D_gene_10000154
novel_mir_270	2	Cotton_D_gene_10036714, Cotton_D_gene_10016627
novel_mir_273	3	Cotton_D_gene_10011395, Cotton_D_gene_10012990, Cotton_D_gene_10000913
novel_mir_274	3	Cotton_D_gene_10032704, Cotton_D_gene_10016308,Cotton_D_gene_10039873
novel_mir_277	1	Cotton_D_gene_10036216
novel_mir_282	5	Cotton_D_gene_10034825, Cotton_D_gene_10032791, Cotton_D_gene_10019765, Cotton_D_gene_10035011, Cotton_D_gene_10026261
novel_mir_286	3	Cotton_D_gene_10032704, Cotton_D_gene_10039873, Cotton_D_gene_10013879
novel_mir_289	1	Cotton_D_gene_10032903
novel_mir_290	1	Cotton_D_gene_10033088
novel_mir_292	2	Cotton_D_gene_10022875, Cotton_D_gene_10013994
novel_mir_296	2	Cotton_D_gene_10022694, Cotton_D_gene_10022695
novel_mir_309	1	Cotton_D_gene_10033485
novel_mir_319	5	Cotton_D_gene_10028854, Cotton_D_gene_10022050, Cotton_D_gene_10012403, Cotton_D_gene_10026100, Cotton_D_gene_10024142
novel_mir_323	7	Cotton_D_gene_10026137, Cotton_D_gene_10036714, Cotton_D_gene_10013795, Cotton_D_gene_10009122, Cotton_D_gene_10009122, Cotton_D_gene_10011063, Cotton_D_gene_10016627
novel_mir_324	3	Cotton_D_gene_10036186, Cotton_D_gene_10013933, Cotton_D_gene_10015879
novel_mir_326	1	Cotton_D_gene_10024886
novel_mir_353	2	Cotton_D_gene_10032704, Cotton_D_gene_10013879

Table S6. *Cont.*

miRNA	Targets	
	Number	Gene ID
novel_mir_372	2	Cotton_D_gene_10039772, Cotton_D_gene_10007827
novel_mir_374	1	Cotton_D_gene_10029444
novel_mir_94	1	Cotton_D_gene_10036376

Note: Only genes with known or putative functions were presented.

Table S8. List of selected miRNAs and the corresponding primers used for real time RT-PCR analysis.

No.	miRNA (sequence)	miR9-RT (5′ → 3′)	miR-S (5′ → 3′)
1	miR5658 (UGUGAUG AUGAAGAUGGAA)	CTCAACTGGTGTCGTGGAGTC GGCAATTCAGTTGAGTTCCATCT	GGGTGTGATGA TGAAGATGGAA
2	miR159 (TTTGGATT GAAGGGAGCTCTA)	CTCAACTGGTGTCGTGGAGTC GGCAATTCAGTTGAGTAGAGCTC	GTTTGGATTGA AGGGAGCTCTA
3	miR172 (AGAATCTT GATGATGCTGCAT)	CTCAACTGGTGTCGTGGAGTCG GCAATTCAGTTGAGATGCAGCA	TGAGAATCTTG ATGATGCTGCAT
4	Cotton 5S	CCGACACTAATGCACCGGAT	CGAGGACTTCC CAGGAGGTC
